# Design and Simulation of a MEMS Control Moment Gyroscope for the Sub-Kilogram Spacecraft

**DOI:** 10.3390/s100404130

**Published:** 2010-04-26

**Authors:** Honglong Chang, Wenlong Jiao, Qianyan Fu, Jianbing Xie, Weizheng Yuan

**Affiliations:** Micro and Nano Electromechanical System Laboratory, Northwestern Polytechnical University, Xi’an, Shaanxi, China; E-Mail: yuanwz@nwpu.edu.cn (W.Y.)

**Keywords:** MEMS, microactuator, control moment gyroscope, attitude control, sub-kilogram spacecraft

## Abstract

A novel design of a microelectromechanical systems (MEMS) control moment gyroscope (MCMG) was proposed in this paper in order to generate a torque output with a magnitude of 10^−6^ N·m. The MCMG consists of two orthogonal angular vibration systems, *i.e.,* the rotor and gimbal; the coupling between which is based on the Coriolis effect and will cause a torque output in the direction perpendicular to the two vibrations. The angular rotor vibration was excited by the in-plane electrostatic rotary comb actuators, while the angular gimbal vibration was driven by an out-of-plane electrostatic parallel plate actuator. A possible process flow to fabricate the structure was proposed and discussed step by step. Furthermore, an array configuration using four MCMGs as an effective element, in which the torque was generated with a phase difference of 90 degrees between every two MCMGs, was proposed to smooth the inherent fluctuation of the torque output for a vibrational MCMG. The parasitic torque was cancelled by two opposite MCMGs with a phase difference of 180 degrees. The designed MCMG was about 1.1 cm × 1.1 cm × 0.04 cm in size and 0.1 g in weight. The simulation results showed that the maximum torque output of a MCMG, the resonant frequency of which was approximately 1,000 Hz, was about 2.5 × 10^−8^ N·m. The element with four MCMGs could generate a torque of 5 × 10^−8^ N·m. The torque output could reach a magnitude of 10^−6^ N·m when the frequency was improved from 1,000 Hz to 10,000 Hz. Using arrays of 4 × 4 effective elements on a 1 kg spacecraft with a standard form factor of 10 cm × 10 cm × 10 cm, a 10 degrees attitude change could be achieved in 26.96 s.

## 1. Introduction

Miniature, low cost, and autonomous spacecrafts have been the focus of NASA since 1992 [1], and will play an increasingly important role in a broad spectrum of planetary, space physics, and earth science missions. On the other hand, microelectromechanical systems (MEMS) are an enabling technology allowing the development of small products. It is widely recognized that MEMS technology, characterized by small size, a light weight, and lower power consumption, should and will have many useful applications in aerospace. In recent years, a variety of microsensors and microactuators have been successfully inserted into aerospace applications [2,3]. One of the major applications of the microactuators is to change the attitude of the miniature spacecraft with a weight of 0.1 to 20 kg.

There are two fundamental ways to control the attitude of spacecrafts, either by applying external torques through technologies such as thrusters [4–16], magnetic torquers [17,18] and Solar sails [19–21], or by changing the angular momentum internally via momentum exchange devices such as reaction wheels (RW) [22,23], control moment gyroscopes (CMG) [24–27], and variable speed control moment gyroscopes (VSCMG) [28–31]. Thrusters are most commonly employed on past and present satellites [4–16]. Their limitations are fuel usage and engine wear. Magnetic torquers work only where there is a magnetic field to react against. Solar sails, which produce thrust as a reaction force induced by reflecting incident light, may be used to make small attitude control and velocity adjustments. These external torques change the total system angular momentum via energy conversion, while the internal torques do not generally change the total system angular momentum.

The working principles of RW, CMG, and VSCMG are very similar. When a torque is exerted on the wheel, an equal and opposite reaction torque is applied to the spacecraft. RWs are electric motor driven rotors made to spin in the direction opposite to that required to re-orient the spacecraft [22,23]. Their limitations are bearing friction and breakdown problems. CMGs own many advantageous properties, such as a large torque amplification capability, big moment storage capability and high agility [26]. CMGs have been employed in many spacecraft missions including the international space station [24,25]. VSCMGs combine positive features of both the single-gimbal CMGs and the RWs, thus adding an extra degree of control to the classical single-gimbal CMG device [28–31].

The conventional CMGs were large in size, mechanically complex, and expensive. Many efforts have been made to obtain mini-CMGs to satisfy the needs of small satellites with a weight of 1–20 kg [26,27]. However these mini-CMGs still used similar technologies to the traditional ones, and they are too big for emerging sub-kg spacecrafts such as pico-satellites or femto-satellites [32–35]. The RWs or CMGs based on MEMS technologies could provide attitude controlling devices with a much smaller size.

The major challenge to realize the RW or CMG through MEMS technologies lies in the miniaturization of traditional momentum wheels. Eunjeong Lee proposed a fully rotating miniature flywheel based on high temperature superconductor technology for nano satellites [36]. Peczalski proposed a micro wheel using a stack of silicon wafers [37], the fabrication of which was somewhat difficult to implement. A pseudo wheel was presented to generate the torque [38,39], the sequence control of which was complicated. Reiter proposed a novel MEMS CMG (MCMG) design concept based on angular vibration instead of angular rotation [40], the torque output of which is projected to reach 2.3 × 10^−12^ N·m. Obviously, this vibration-based miniature CMG eliminates the need for advanced bearings. It is quite a promising attitude control technology. However, the magnitude of the torque was too small to change the attitude of a sub-kg spacecraft. In this paper, we designed a MCMG in detail using a feasible micromachining process flow. Furthermore, the maximum torque output was largely improved, which would bring forth possible applications in sub-kg spacecrafts.

## 2. Working Principle of the MCMG

**Figure 1.**
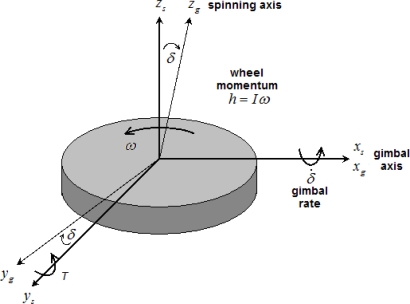
Schematic of a single gimbal CMG.

The working principle of the MCMG is similar, yet precisely opposite, to that of conventional MEMS Coriolis vibratory gyroscopes (CVG). MCMGs uses Coriolis effect to influence the outside world, while MEMS CVGs use Coriolis effect to sense the outside world. The proposed MCMG is actually a single gimbal CMG. For development equations of the torque output, two reference frames, *i.e.,* gimbal reference frame (*x_g_*, *y_g_*, *z_g_*) and substrate reference frame (*x_s_*, *y_s_*, *z_s_*) are defined as shown in Figure 1. The two reference frames have a common origin, which locates at the center of the spinning disc. When the gimbal angle *δ* is zero, the two frames coincide with each other. The spinning disc is assumed as a rigid body, which is symmetric about its spinning axis *z_g_* in the gimbal reference frame. In the MCMG, the spinning disc or rotor rotates with an angular velocity *ω* about *z_g_* relative to the gimbal reference frame. Therefore the angular momentum of the rotor in the substrate reference frame is defined as following:

(1) h=Iω

where *I* is the inertial moment of the spinning disc about *z_g_*.

If a rotation is applied to the spinning disc about *x_g_* with a precession rate of *δ̇* in the substrate reference frame, an output torque *T*, which is perpendicular to the directions of *ω* and *δ̇*, will be generated about torque output axis *y_g_* [26,40].

(2) $$T=h\;\dot{\delta }$$

In the proposed MCMG, angular sinusoidal vibrations substitute the angular rotations. The angular displacements of rotor and gimbal in corresponding frames can be expressed as following:

(3) $$a_{r}(t)=A_{r}\;\text{sin}(2\pi \;\mathrm{ft})$$

(4) $$\delta (t)=A_{g}\;\text{sin}(2\pi \;\mathrm{ft})$$

where *A_r_*, *A_g_* is the angular vibration amplitude of the rotor and the gimbal, respectively; *f* is the resonant frequency of rotor and gimbal. Then the angular velocities of rotor and gimbal can be generated as following:

(5) $$\omega \;(t)={\dot{a}}_{r}(t)=2\pi {\mathrm{fA}}_{r}\;\text{cos}(2\pi \;\mathrm{ft})$$

(6) $$\dot{\delta }(t)=2\pi {\mathrm{fA}}_{g}\;\text{cos}(2\pi \;\mathrm{ft})$$

Substituting Equation (1), Equation (5), and Equation (6) into Equation (2), the output torque can be described as following:

(7) $$T=I{\left(2\pi \;f\right)}^{2}A_{r}A_{g}\;{\text{cos}}^{2}(2\pi \;\mathrm{ft})$$

It is obvious that the amplitude of the output torque is proportional to the inertial moment of spinning disc *I*, resonant frequency *f*, and the amplitude of vibration. This is the theoretical basis for the further structure design of MCMG.

## 3. Structure and Process Flow for MCMG

**Figure 2.**
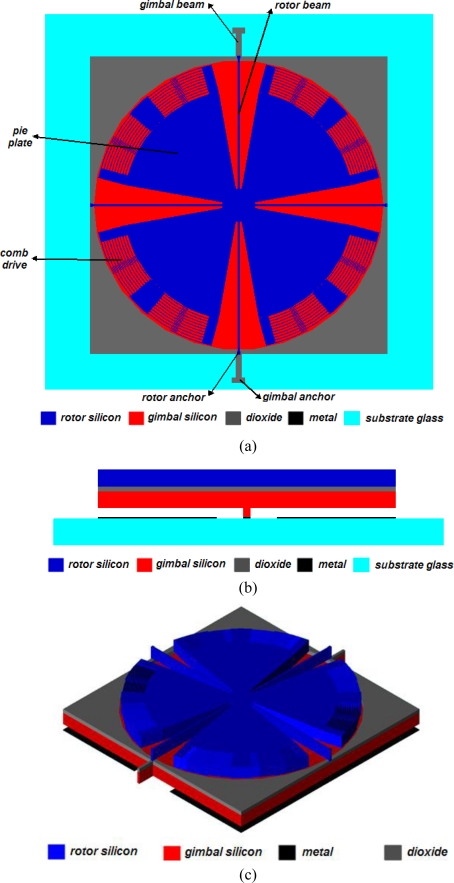
(a) Top view of MCMG. (b) Cross section of MCMG. (c) Solid model of MCMG. (The figures are not to scale).

MCMG consists of two orthogonal angular vibration systems, *i.e.,* rotor and gimbal angular vibration system. A proposed design scheme for MCMG is shown in Figure 2. The rotor is an in-plane rotary electrostatic comb drives with four clamped straight beams as suspension (Figure 2a), which is a mature design to realize the angular vibration of the rotor [41]. The parallel plate electrostatic actuators have been successfully used in many applications [42]. In the proposed MCMG, the gimbal is supported by two beams anchored on the substrate, and driven by electrostatic parallel plate actuators (Figure 2b). The rotor and gimbal are connected through four rotor suspension beams. The rotor is always resonated by the in-plane rotary electrostatic comb drives. Thus, when the gimbal is excited by the electrostatic parallel plate actuators, the vibration will transfer to the rotor, and cause it to vibrate out of the plane about the same axis too. Then, a torque based on the Coriolis effect will be generated and applied on the substrate.

**Figure 3.**
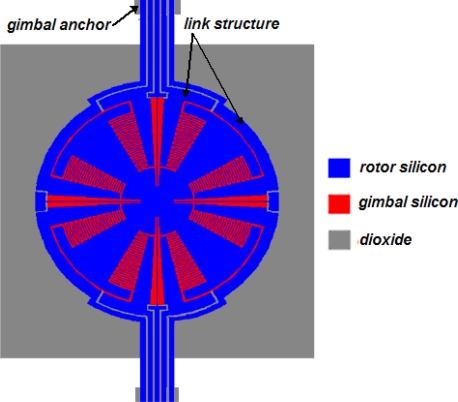
The designed link structure to supply electronic voltage.

Wire pads for comb drives are usually located at fixed combs. This can, however, not be used for MCMGs. The vibration of the SOI sandwich will break off the wires. To solve this problem, the ‘link structure’ (Figure 3) is proposed. It extends from ‘fixed combs’ to ‘gimbal anchors’. The wire pads are placed at stationary ‘gimbal anchors’, and provide electrical voltages to comb drives through the ‘link structure’.

**Figure 4.**
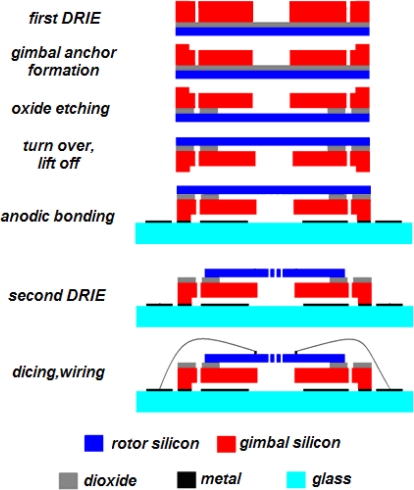
The proposed process flow for the MCMG.

A process flow is proposed to realize such a MCMG structure as shown in Figure 4. The major steps in the process flow are the bonding between a silicon-on-insulator (SOI) wafer, and a glass wafer. Both the rotor and the gimbal are fabricated on the SOI wafer, while the gimbal is actuated by the electrostatic parallel plate actuators between the SOI wafer and the metal on the glass wafer. The fabrication sequences can be performed as following. Firstly, patterns such as damping holes in the SOI handle layer are formed by the first deep reactive ion etching (DRIE). Then, the SOI layer is wet etched to form the ‘gimbal anchors’. Now etchants can flow through the damping holes, and the oxide is etched off. Secondly, metal is formed on the SOI device layer to provide voltages on the comb drives, and metal electrodes are formed on the glass by a lift-off process. Then, the glass and the SOI layer are bonded together. Thirdly, the second DRIE defines structures including the rotor and comb drives in the SOI device layer. The final steps are dicing and wiring.

This process flow has three advantages at least. In contrast with the surface process [40], this bulk process increases the output torque and ensures the rigidity of structure. Secondly, the removal of oxide is placed between two DRIE steps, so that footing can be avoided in the second DRIE, which is very helpful to protect the bottom geometry of those finer structures such as comb drives formed in the second DRIE. Thirdly, the rotor-defining DRIE is carried out after the removal of oxide. Thus, the stiction between the silicon layers can be avoided.

## 4. Behavior Simulations of MCMG

**Figure 5.**
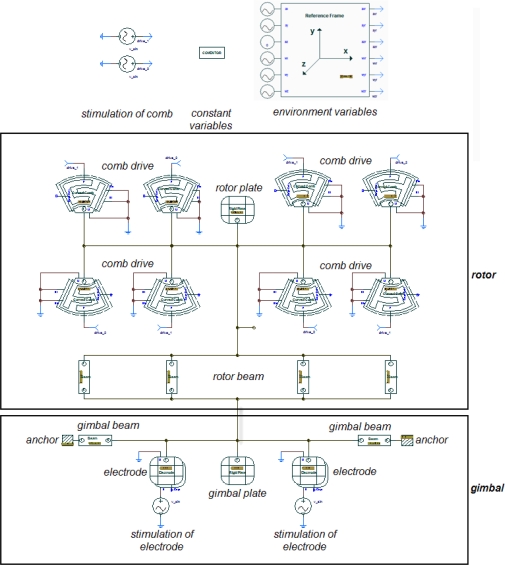
The system level model of MCMG established in Architect.

**Figure 6.**
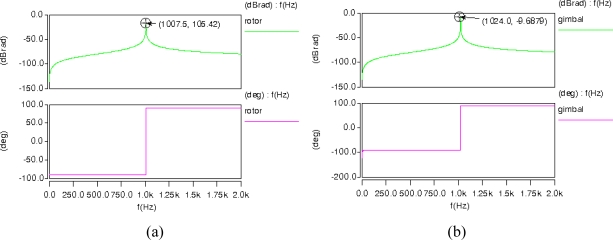
(a) AC analysis results of the rotor. (b) AC analysis results of the gimbal.

Based on the aforementioned design scheme, we gave a group of major parameters with their feasible values as shown in Table 1. The size of a MCMG was about 1.1 cm × 1.1 cm × 0.04 cm, and the weight was about 0.1 g. We used a popular commercial MEMS design tool, Architect from Coventor Inc., to model and simulate MCMG. Various parametric components such as curved comb drives, plates and beams, were connected to form the system level model of MCMG as shown in Figure 5. AC analysis results showed that the resonant frequency of rotor and gimbal was 1007.5 Hz and 1024.0 Hz, respectively (Figure 6). Thus, resonant frequencies of the rotor and the gimbal were well matched. Transient analysis was executed after the AC analysis. In order to make electrostatic forces and driving voltages have a linear relationship, the rotor and gimbal were bilaterally driven by sinusoidal biased voltages. The voltages applied on the rotor’s bilateral sides were *V*_1_ and *V*_2_, and the voltages applied on the gimbal’s two electrodes of the parallel plate actuator were *V*_3_ and *V*_4_. The expressions of the voltages are listed in Table 1.

**Figure 7.**
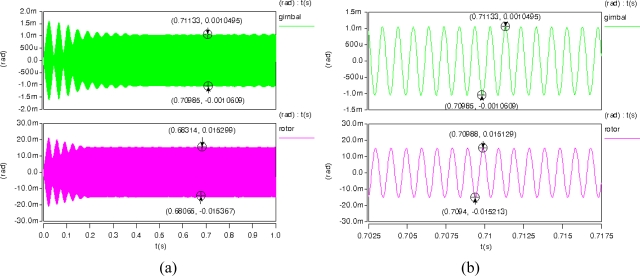
(a) Transient analysis results of the MCMG. (b) A closer view of the results.

Transient analysis results of the MCMG are shown in Figure 7. The results show that the maximum angular displacements of the rotor and the gimbal were 0.0151 *rad* (0.87°) and 0.00104 *rad* (0.06°), respectively. According to Equation (7), the maximum torque output of the MCMG was 2.5 × 10^−8^ N·m. The rotor’s kinetic energy in the MCMG can be calculated by the equation as following.

(8) $$E_{r}=\frac{1}{2}I\omega^{2}(t)=2I\pi^{2}f^{2}A_{r}^{2}\;{\text{cos}}^{2}(2\pi \;\mathrm{ft})$$

**Figure 8.**
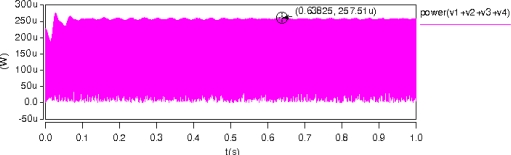
The power of voltage sources in a MCMG.

The maximum instantaneous energy that the rotor sustained is just the maximum kinetic energy of the rotor. According to Equation (8), it is about 1.8 × 10^−7^ J. The power of a single MCMG can also be obtained from the transient analysis results. The total power of four voltage sources, *i.e., V*_1_, *V*_2_, *V*_3_ and *V*_4_, was about 0.26 mW as shown in Figure 8. Ideally, the instantaneous maximum power of a MCMG is equal to the maximum power consumption of the sources without energy loss.

The output torque of the designed MCMG can be enhanced further in several ways. It is very efficient to enhance the torque output by increasing the resonant frequency, since the torque is proportional to square of the frequency. As for the current design, if we improved the resonant frequency from 1000 Hz to 10,000 Hz, then the torque output could reach a magnitude of 10^−6^ N·m. Increasing inertial moment of the rotor means to augment the size of the whole MCMG. However, the size of a chip is usually restricted by the corresponding micromachining process. The layout area for the MCMG with 3 microns as the critical dimension is usually limited to 1.0 cm × 1.0 cm approximately. Another way is to increase the driving voltages to enlarge the angular vibration amplitude of both rotor and gimbal. However, the pull-in phenomenon of parallel plate capacitor actuators [43,44] needs to be avoided when increasing voltages.

## 5. Application Issues of the MCMG

**Figure 9.**
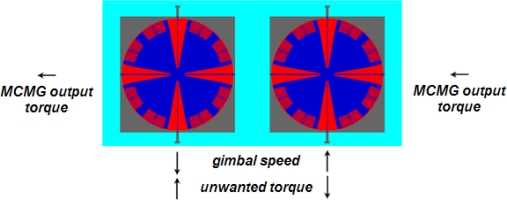
Cancellation of the parasitic torque by the MCMG pair with an opposite phase.

In order to meet the requirements of practical attitude control, parasitic torque and fluctuations of torque output for MCMG need to be eliminated or smoothed. The parasitic torque is generated due to change of the gimbal momentum. A configuration of two-MCMG pairs with an opposite phase can cancel the parasitic torque effectively (Figure 9). Unlike constant torque generated by conventional CMGs, the torque of a vibratory MCMG fluctuates in nature, and follows sine wave. According to Equation (7), two MCMGs having a phase difference of *π* / 2 will solve this problem and make output torque of the MCMGs stable.

According to Equation (7), we assume that the output torques of two MCMGs are *T*_1_ = *A* cos^2^ *ωt* and *T*_2_ = *A* cos^2^ (*ωt* + *π* / 2), respectively. So the sum of output torques is

(9) $$T_{1}+T_{2}=A{\text{cos}}^{2}\;\omega t+A{\text{cos}}^{2}(\omega t+\pi /2)=A$$

where *A* = *I* (2*πf*)^2^ *A_r_A_g_*.

**Figure 10.**
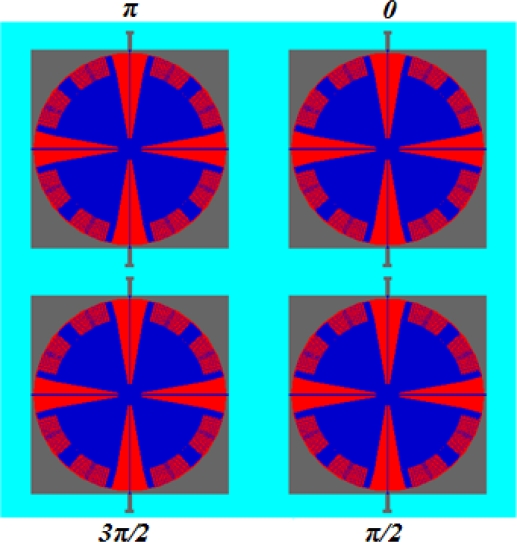
The array configuration with four MCMGs as an element.

All in all, in order to solve the two problems mentioned above, an array configuration using four MCMGs as an effective MCMG element, in which the torques are generated with a phase difference of *π* / 2 between every two MCMGs (Figure 10), is proposed to stabilize the overall torque at the array level and eliminate the unwanted parasitic torques. Thus, an effective MCMG element can output a maximum torque of 5 × 10^−8^ N·m.

**Figure 11.**
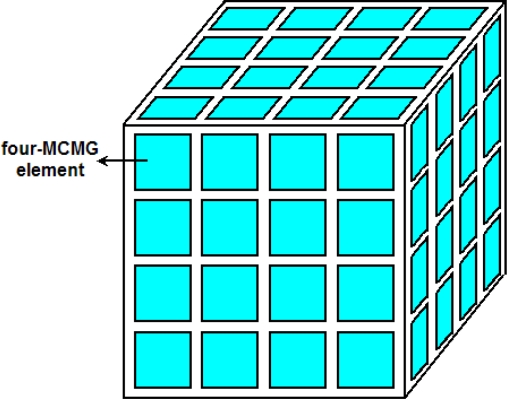
The array configuration of MCMG elements on a spacecraft.

A 1 kg cubic spacecraft with a standard form factor of 10 cm × 10 cm × 10 cm [33] is taken as an example carrier to discuss the application issues. As shown in Figure 11, each of its six faces will be assigned 16 MCMG elements (a 4 × 4 MCMG elements array), which can generate a torque of 0.8 × 10^−6^ N·m. Then the spacecraft can change its attitude around three axes in Cartesian coordinate system. Using such a configuration, a 10-degree attitude change, a five-degree acceleration phase and a five-degree deceleration phase [45], about one of the three central axes of the spacecraft will take 26.96 s. The average slew rate is about 0.37°/s, and it is suitable for sub-kilogram spacecrafts.

## 6. Conclusions

In this paper, a novel concept of MEMS control moment gyroscope was designed and simulated, and a possible process flow was also presented. The performance of the MCMG was projected to have a torque output of 2.5 × 10^−8^ N·m, even larger to a magnitude of 10^−6^ N·m. The proposed four-MCMG array configuration with a phase difference of 90 degrees between every two MCMGs was proved very effective to null out the parasitic torque and smooth the output torque. Through a proper configuration, the MCMG could be used to generate a torque output that is big enough to change the attitude for the sub-kg spacecraft.

## Figures and Tables

**Table 1.** Major parameters for the MCMG.

**Parameters**	Length of gimbal	Thickness of gimbal	Length of gimbal beam	Width of gimbal beam	Thickness of dioxide layer
***Values***	*9,000 μm*	*299 μm*	*700 μm*	*101 μm*	*5 μm*
**Parameter**	Length of rotor beam	Width of rotor beam	Thickness of rotor	Inner diameter of pie plate	Outer diameter of pie plate
***Values***	*3800 μm*	*64 μm*	*80 μm*	*500 μm*	*3460 μm*
**Parameter**	Length of pie plate	Comb finger length	Comb finger width	Comb finger gap	Comb finger overlap
***Values***	*70°*	*14°*	*4 μm*	*3 μm*	*3°*
***Rotor voltage***	*V*_1_ = 25 + 25 sin(2*π ft*) *V*_2_ = 25 + 25 sin(2*π ft* + *π*)				
***Gimbal voltage***	*V*_3_ = 50 + 50 sin(2*π ft*) *V*_4_ = 50 + 5 sin(2*π ft* + *π*)				
